# Emissions of microplastic fibers from microfiber fleece during domestic washing

**DOI:** 10.1007/s11356-016-7703-0

**Published:** 2016-09-22

**Authors:** U. Pirc, M. Vidmar, A. Mozer, A. Kržan

**Affiliations:** 1 Gimnazija Vič, Tržaška 72, Ljubljana, Slovenia; 2 National Institute of Chemistry, Laboratory for Polymer Chemistry and Technology, Hajdrihova 19, Ljubljana, Slovenia

**Keywords:** Textile, Fiber, Microplastics, Washing, Pollution, Polyethylene terephthalate

## Abstract

Microplastics are found in marine and freshwater environments; however, their specific sources are not yet well understood. Understanding sources will be of key importance in efforts to reduce emissions into the environment. We examined the emissions of microfibers from domestic washing of a new microfiber polyester fleece textile. Analyzing released fibers collected with a 200 μm filter during 10 mild, successive washing cycles showed that emission initially decreased and then stabilized at approx. 0.0012 wt%. This value is our estimation for the long-term release of fibers during each washing. Use of detergent and softener did not significantly influence emission. Release of fibers during tumble drying was approx. 3.5 times higher than during washing.

**Electronic supplementary material:**

The online version of this article (doi:10.1007/s11356-016-7703-0) contains supplementary material, which is available to authorized users.

## Introduction

Plastics were first noticed in oceans in the 1970s (Buchanan 1971; Carpenter et al. 1972; Carpenter and Smith 1972) when plastic production was still far below current levels. More recently, attention of the wider public was caught by the discovery of the north Pacific gyre “garbage patch” by Charles Moore (Moore and Phillips 2012). In the last decade, microplastics (MP), plastic particles smaller than 5 mm (Andrady 2011; Ivar do Sul and Costa 2014), have been gaining attention (Law and Thompson 2014) and a rapidly increasing body of research is now available showing that MP are found in all strata of the marine environment as well as in freshwater environments (Dris et al. 2015; Klein et al. 2015; Gallagher et al. 2015) and even in foodstuffs (Liebezeit and Liebezeit 2013, 2014).

MP are of similar sizes as plankton and can be easily ingested by organisms (Wright et al. 2013). The chemical structure of plastics supports adsorption of non-polar persistent organic pollutants (POPs) (Hirai et al. 2011; Rochman et al. 2013), and the effect is augmented by the increase of the surface-to-volume ratio with decreasing particle sizes. When MP are ingested, the leaching of adsorbed pollutants and additives could be a source of toxic substances influencing the organisms and entering into the food web leading all the way to humans (Koelmans et al. 2013; Rochman et al. 2013; Van Cauwenberghe and Janssen 2014; Rochman et al. 2015).

Sources of MP are known only generally as follows: they emerge from direct use of small particles (primary MP) or from fragmentation of larger plastic debris (secondary MP). Thompson et al. (2004) reported that microfiber concentrations in historical sea-surface water samples correlated with the production volume of synthetic fibers in manufacturing. A prioritization of sources by Verschoor et al. (2014) put a high (7/10) score to textiles and garments made from synthetic materials, which shed fibers during washing and use. In the past, degradation of textiles was studied to understand the limits on useful fabric life (Slater 1986) or in forensics (Watt et al. 2005; De Wael 2010) while emissions into the environment were not a concern. Browne et al. (2011) were the first to identify washing as a source of pollution with plastic fibers. They reported, “a single garment can shed more than 1900 fibers per wash and that all garments released more than 100 fibers per liter of effluent;” however, significant information on the textiles used and the experimental methodology is not reported. Dubaish and Liebezeit (2013) reported a release of 0.033–0.039 wt% fibers from a polyester garment per washing although experimental conditions are not given. A study by the Norwegian Environment Agency (Sundt et al. 2014) estimated the annual fiber release from laundries and households in Norway at 100 and 600 t, respectively, however identified the need for better data as an important knowledge gap. A study by Petersson and Roslund (2015) shows that yarn and textile type combined with usage most affect fiber release during washing. Habib et al. (1996) as well as Zubris and Richards (2005) reported synthetic fibers as an indicator of municipal sewage sludge use in soils, indicating fiber presence in wastewaters as well as spreading routes.

The goal of our study was to obtain currently unavailable mass-based data on the release of fibers during washing of a typical textile that would enable an estimate of the cumulative mass of fibers released into the environment from this source. We also studied the effect of washing detergent and softener on the release.

## Materials and methods

Textiles used in the experiments were six identical fleece blankets (120 × 70 cm) purchased for the purpose (Supp. Inf. Fig S1). The blankets were bright red in order to facilitate fiber identification. The average blanket weighed 320 g. FTIR (Perkin Elmer, Spectrum One), scanning electron microscopy (SEM, Carl Zeiss supra 35VP), and stereomicroscopy (Leica DMS 1000) were used.

The washing machine used was a brand new front-loading Bosch model Maxx7 VarioPerfect. Using a new machine reduced the problem of contamination by residual fibers in the machine. Detergent Ariel (Procter & Gamble, France) and fabric softener/conditioner Silan (Henkel, Austria), both commercial products were used.

Collection of released fibers was performed by filtering wastewater from the washing machine using an external custom-built filtration setup with a removable stainless steel filter (disk-shaped, 85 mm diameter) with 200 × 200 μm openings (Supp. Inf. Fig S2). The filter with the collected fibers was removed from the setup and air-dried to a constant mass in a dust-free environment prior to weighing. Relative fiber release was calculated as Δm/% = *m*_(f)_/*m*_o(b)_ × 100 where *m*_(f)_ equals the mass of fibers collected on the filter, and *m*_o(b)_ signifies the initial blanket mass.

Washing experiments were carried out using the SuperQuick15 program (duration 15 min, temperature 30 °C, spinning 600 rpm). Experiments in which detergent and/or fabric softener was used were carried out with the addition of 10 mL of each in a fashion specified by the appliance producer. Prior to each set of 10 experiments, the empty machine was cleaned by two wash cycles using a more rigorous program (105 min., 60 °C, 1200 rpm). In the first cleaning cycle, 150 g of citric acid was added to the washing compartment. The wastewater from the cleaning cycles was filtered to monitor that the machine was clean. In all cases, the collected residue on the filters was negligible (max. 0.1 mg (0.00034 ‰)). After each washing experiment, the blanket was tumble-dried in a Whirlpool (AWZ865) drying machine at 40 °C for 18 min. Fibers collected on the built-in filter (openings 180 × 180 μm, Supp. Inf. Fig. S3) were weighed.

## Results and discussion

**Fig. 1:**
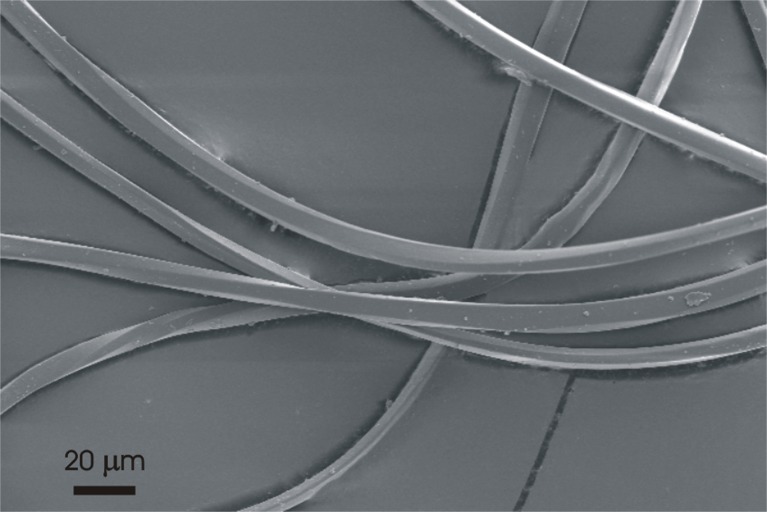
Scanning electron micrograph of PET microfibers

The material of the blankets was identified by FTIR as polyethylene terephthalate (PET) polyester. The fabric of the deep-red fleece blanket consisted of a ground textile weft-knitted fabric made from texturized, delustered polyester multifilament yarn with a fine filament titer of approximately 2.5 dtex (diameter approx. 15 μm). The loop piles for the double-sided plush consisted of texturized PET microfiber (fiber diameter 10 μm—approx. 1 dtex) multifilament yarn with at least 200 filaments, which was cut or raised to a loop height of approximately 10 mm. The structure is shown in Supp. Inf. Fig. S4. A SEM micrograph of the microfiber is shown in Fig. 1.

Three series of washing experiments were carried out: (1) without additions, (2) with detergent, and (3) with detergent and fabric softener. In each series, two blankets were separately washed and dried 10 times (Supp. Inf. Fig. S5).

**Fig. 2:**
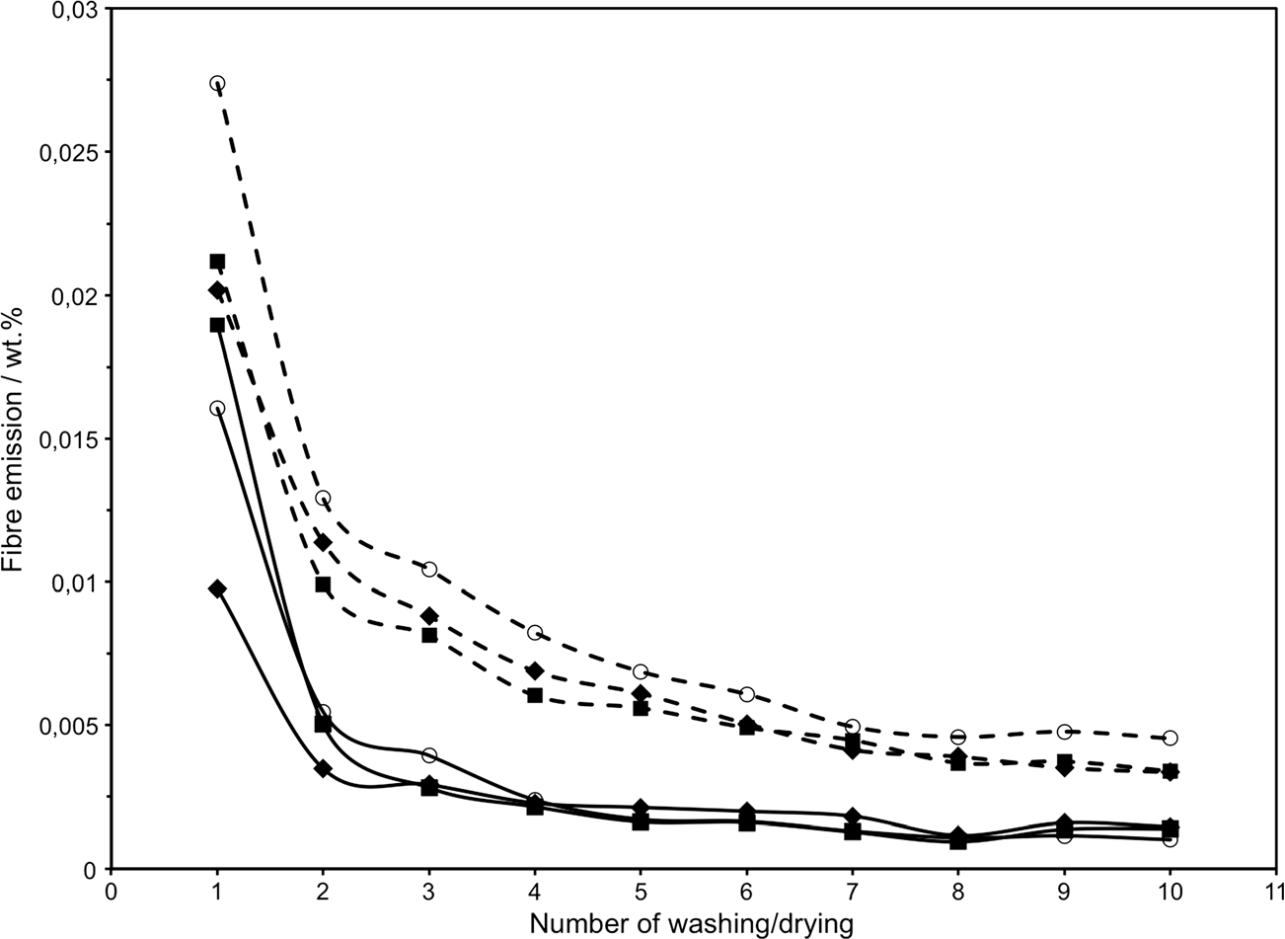
Average relative quantities of microfibers released during successive washing (*solid lines*) and drying (*dashed lines*) of PET microfiber blanket. *Empty circle* indicates without additives, *filled diamond* with detergent, and *filled square* with detergent and fabric softener

Plots in Fig. 2 (Table 1) show the average relative release of fibers. Results of the first washing varied the most, 0.008–0.021 wt% fibers released, which is attributed to differences between the as-purchased blankets. The differences quickly decreased during subsequent washing cycles. The two parallel experiments done in each series were generally in good agreement (detailed results Supp. Inf. Table S1, Fig. S6). Fiber release stabilized during the last few washing experiments. The average values from cycles 8, 9, and 10 were taken as an estimate for a stable release expected on a long-term basis: 0.00108 wt% (no additives), 0.00140 wt% (detergent), and 0.00124 wt% (detergent + softener). These certain, however low, differences indicate that additives are not a main factor in fiber release but rather a mechanical stress. The average of all three series is 0.00127 wt%. By using a rougher filter than the paper filter used by Browne et al. (2011), we prevented clogging and were able to evaluate the effect of washing additives.

**Table 1:** Average relative fiber emissions in weight % in 10 consecutive washing/drying experiments of a polyethylene terephthalate microfiber blanket without additives, using detergent, and using detergent and fabric softener. Each value is based on two parallel experiments

Experiment	No additive		Detergent		Detergent and softener	
	Washing	Drying	Washing	Drying	Washing	Drying
1	0.01606	0.02740	0.00976	0.02017	0.01895	0.02117
2	0.00547	0.01291	0.00348	0.01137	0.00503	0.00992
3	0.00394	0.01043	0.00293	0.00882	0.00282	0.00814
4	0.00239	0.00823	0.00228	0.00689	0.00215	0.00604
5	0.00171	0.00687	0.00213	0.00611	0.00163	0.00560
6	0.00165	0.00608	0.00199	0.00505	0.00161	0.00491
7	0.00131	0.00495	0.00182	0.00414	0.00128	0.00448
8	0.00108	0.00459	0.00116	0.00390	0.00095	0.00368
9	0.00115	0.00478	0.00159	0.00351	0.00137	0.00373
10	0.00102	0.00454	0.00146	0.00336	0.00139	0.00339
Average of exp. 8, 9, 10	0.00108	0.00464	0.00140	0.00359	0.00124	0.00360

The wastewater after filtering contained no visible fibers. To verify the efficiency of a filter with relatively large openings (200 μm) relative to fiber thickness (10 μm), we further filtered two samples of effluent water using a paper filter with 2–3-μm pores. The quantity of collected fiber fragments was very low (we estimate a maximum of several % of the released fibers), but we were not able to quantify it due to large volumes and clogging problems. The fragments were mainly in the 20–200 μm range (Supp. Inf. Fig. S7) with very few long fibers (max. approx. 700 μm). These results show that installation (and maintenance) of a relatively simple and robust filter could prevent most of the emissions.

To verify our results, we washed a five-year-old PET fleece jacket. Microfiber release was 0.00111 wt% (no additive), 0.00123 wt% (detergent), and 0.00136 wt% (detergent + softener) giving an average of 0.00123 wt%. This result is in good agreement with our experimental estimate for long-term release obtained with the blankets, confirming it as an acceptable long-term release value.

We attempted to correlate the mass of the fibers released to their number, however found it impossible to separate the intertwined fibers (Supp. Inf. Fig. S8). We were however able to disentangle some fibers; the range of lengths was 0.3–25.0 mm, with an average length of 5.3 mm; however, the average length may be underestimated due to the particular difficulty of disentangling long fibers. A number of released fibers were very lengthy (up to 25 mm) even after several washings, which is most likely a function of the plush fabric. These lengths indicate disentanglement of full-length fibers covering piles on both sides of the fabric (approx. 10 mm each) and the part fixed in the ground textile. Among all inspected fibers, only one filament from the ground textile (2.5 dtex) was observed while all others originated from pile fibers (1 dtex). Using the average fiber length (5.3 mm), a 1-dtex diameter (1 g per 10.000 m), and the 0.0012 wt% release, we can calculate that a 500-g piece of fabric will release 6 mg of fibers or 11.3 × 10^3^ fibers (although this number may be overestimated due to a likely underestimation of the average fiber length).

Although fibers released during spin-drying are not released into the wastewater, we monitored the quantities (Table 1, Supp. Inf. Table S1). Release of fibers during drying was in all cases higher by an approximate factor of 3.5 than the release during washing. The plot of average releases in Fig. 2 indicates that the long-term release value was not yet reached since values continue trending lower. This assumption was confirmed by the results obtained with the old fleece garment, which gave releases of 0.00111, 0.00103, and 0.00123 wt% during the three drying cycles. The average of 0.00112 wt% is significantly lower than the average 0.00394 wt% we obtained with the blanket.

Our results confirm findings of previous studies indicating fiber release during washing and support the numerous reports of synthetic fibers found in natural marine and freshwater habitats (Thompson et al. 2004; Klein et al. 2015; Gallagher et al. 2015) as well as in organisms (Rochman et al. 2015). The estimated number of fibers released in our experiments (even when taking into account a possible overestimation) is significantly higher than the 1900 fibers per garment/washing reported by Browne et al. (2011) but is much lower than predicted by Bruce et al. (2016)—up to 250 × 10^3^ fibers per garment/washing. It is however significant that we used the 1-dtex mass/fiber-length value which we consider appropriate as opposed to Sundt et al. (2014) and Bruce and al. (2016) using the 300-dtex value. Our weight percent release ratios are significantly lower than the 0.039 wt% reported by Dubaish and Liebezeit (2013) and the very broad range (0.007–0.216 wt%) reported by Bruce et al. (2016) for new garments and front-loading washing machines. Bruce et al. (2016) used two filters (333 and 20 μm) which can attribute for only part of the difference as the smaller mesh collected a minor part of the total fiber release. Partially, the very mild washing conditions used in our experiments probably lead to a conservative fiber release estimate. However, the much longer fibers released in our experiments, 5.3 mm compared to 0.7 mm by Bruce et al. (2016) who used fleece jackets, strongly support the conclusions of Petersson and Roslund (2015) who concluded that fabric structure is the most important factor influencing fiber release. As we are still collecting the first sets of fiber release data, we will need to establish in more detail the influence of washing conditions (e.g., temperature, duration, load size) and fabric properties (fiber type and material, fabric structure, etc.) in order to come to more reliable estimates of the quantitative extent of this type of pollution.

A key result of this study is the indication that fibers are emitted throughout the lifetime of the garment. The importance of our estimated 0.0012 wt% of loose fibers released into the wastewater during each washing lies in the cumulative effects. We performed a rough assessment of emissions for a northern climate with the following assumptions: each resident has one polyester blanket (small size 350 g) washed four times a year and one fleece jacket (500 g), washed eight times a year. Based on our results (0.0012 wt% loss per washing), the mass of released fibers corresponds to 4.5 mg for the blanket and 6.5 mg for the jacket resulting in 70 mg of microfibers released annually per person. For Slovenia with just over 2 million inhabitants, this leads to emissions of approximately 144 kg a year. We believe these are conservative estimates since no new items (with an initially higher release) were considered, and the average person is most likely to own more items made of synthetic fibers (sports clothing, gloves, caps, pet items, etc.). Considering a material density of 1.38 g/cm^3^ and a fiber diameter of 10 μm, we can calculate that this quantity has a surface of 41,700 m^2^. Although it was already shown that the majority of fibers released during washing is removed in wastewater treatment plants where these are used (Talvitie et al. 2015; Mintenig et al. 2014) and despite PET absorbing lower quantities of POPs than polyolefins (Wright et al. 2013), we nevertheless believe that the large specific surface of microfibers qualifies this form of microplastics as an important class with a notable contribution to the overall problem of pollutants carried by microplastics (Rios et al. 2007).

## Conclusions

Results confirm domestic washing of textiles and garments as a constant and widespread source of plastic microfiber emissions into the environment. We estimate that in the case of a long-fiber polyester plush fleece, 0.0012 wt% of loose microfibers is released into wastewaters during every washing. The effect of detergent and fabric softener use is relatively small. The weight-based quantification of emissions should complement published particle-number reports and help in the assessment of cumulative emissions and potential effects. Our results clearly point out that cumulatively large quantities of microplastics are released into the environment from this source.

## Electronic supplementary material

ESM 1 (DOCX 61.4 mb)
