# Supplementary material for: Emissions of microplastic fibers from microfiber fleece during domestic washing
Source: Environ Sci Pollut Res Int. 2016 Sep 22;23(21):22206–11. doi: 10.1007/s11356-016-7703-0 (PMC5099352; doi:10.1007/s11356-016-7703-0)
Supplement: Supplementary file 1 — (DOCX 61.4 mb) [file 11356_2016_7703_MOESM1_ESM.docx]

Supporting Information for

Emissions of Microplastic Fibers from Microfiber Fleece during Domestic Washing

Urša Pirc ^a^, Maja Vidmar ^a^, Alenka Mozer ^a^, Andrej Kržan ^b^^[[1]](#footnote-1)^

^a^ Gimnazija Vič, Tržaška 72, Ljubljana Slovenia

^b^ National Institute of Chemistry, Laboratory for Polymer Chemistry and Technology, Hajdrihova 19, Ljubljana, Slovenia


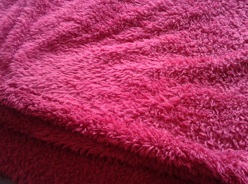

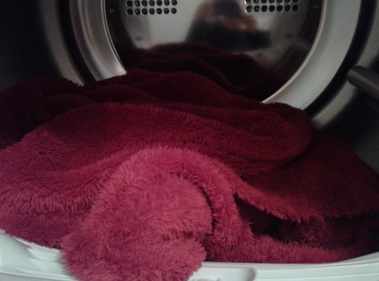


Figure S1. Photo of two blankets used in experiments.


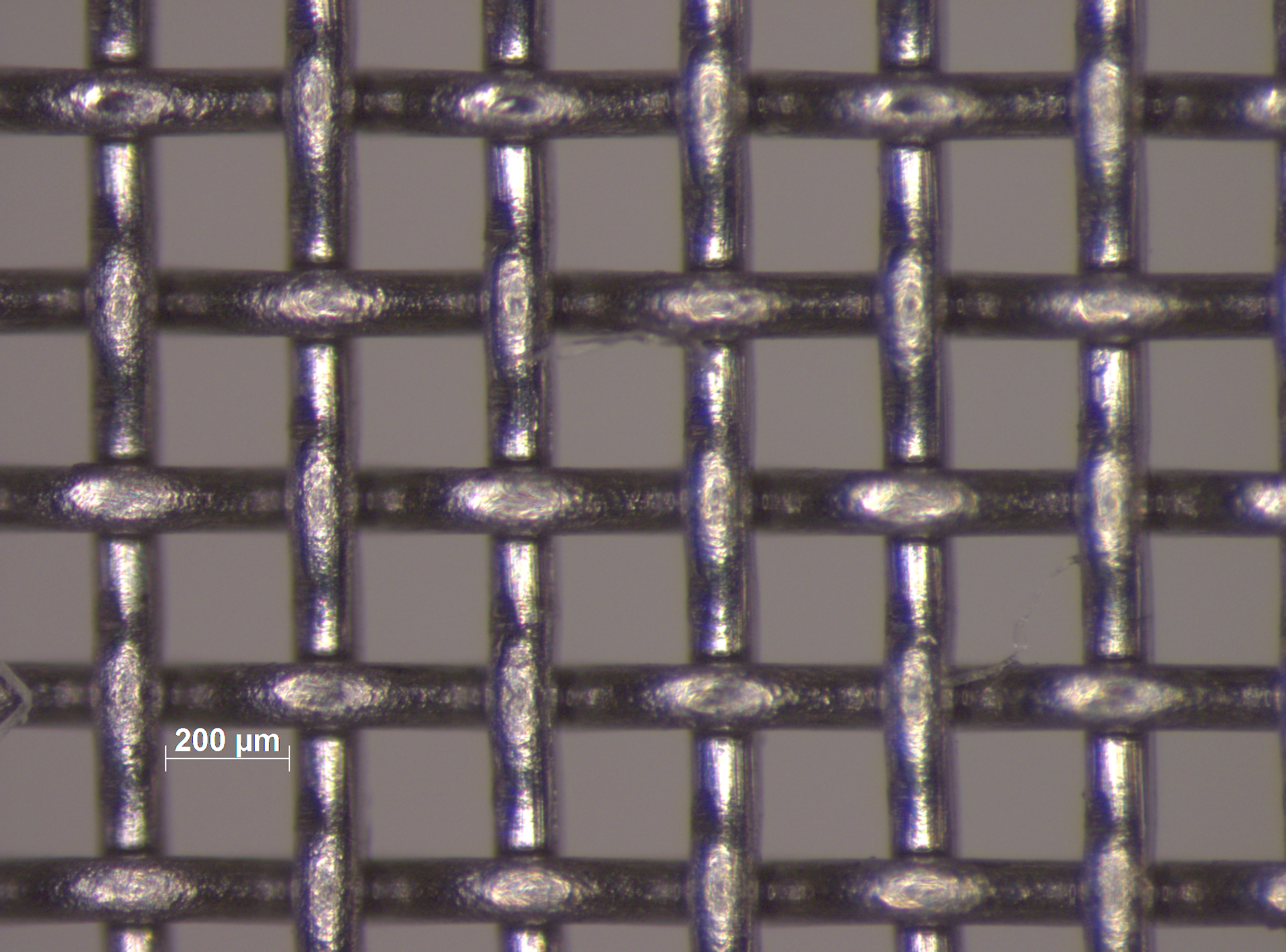


Figure S2. Stereo micrography of stainless steel filter used in experiments


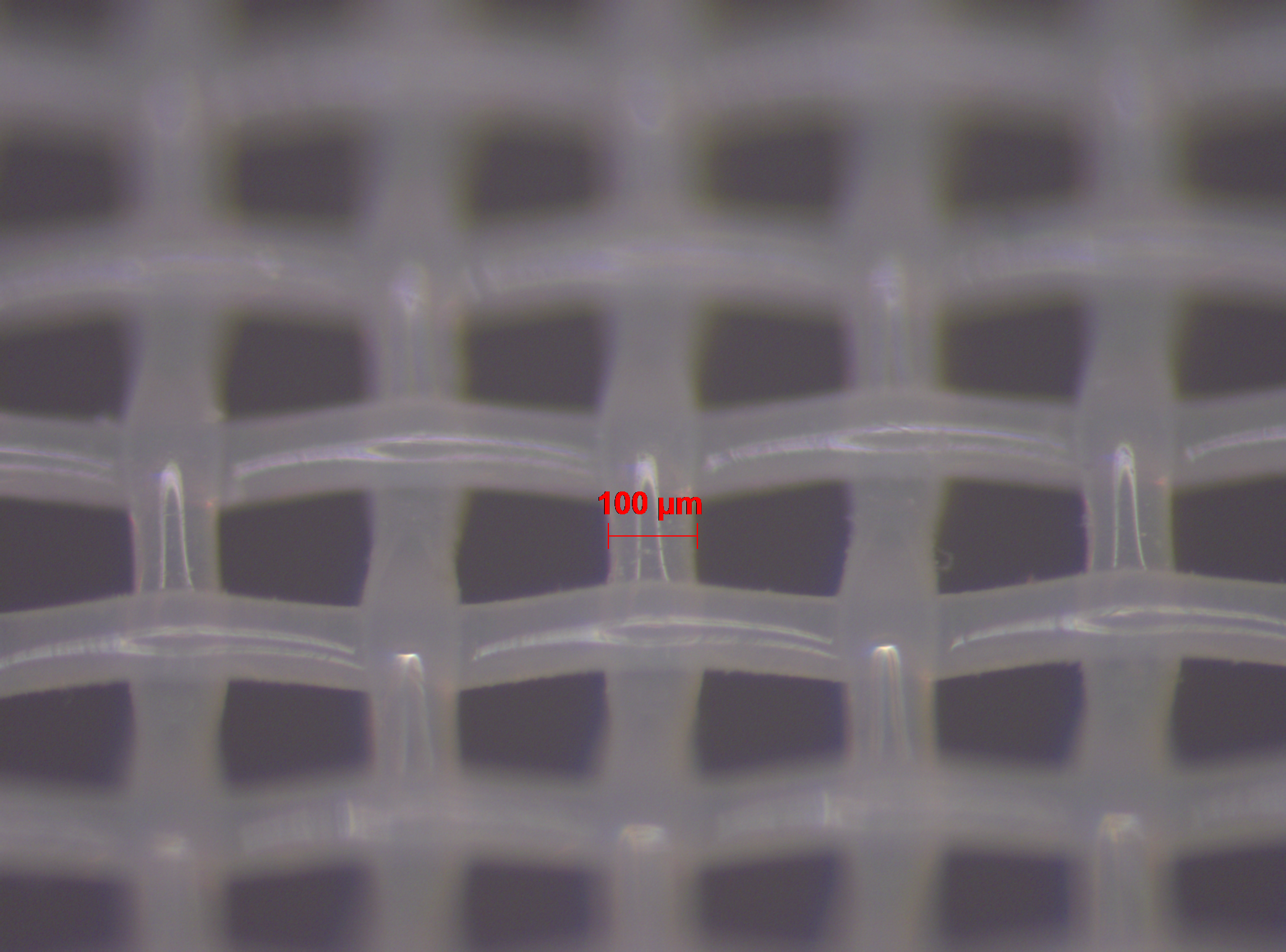


Figure S3. Stereo microscopy images of plastic filter in tumble dryer.


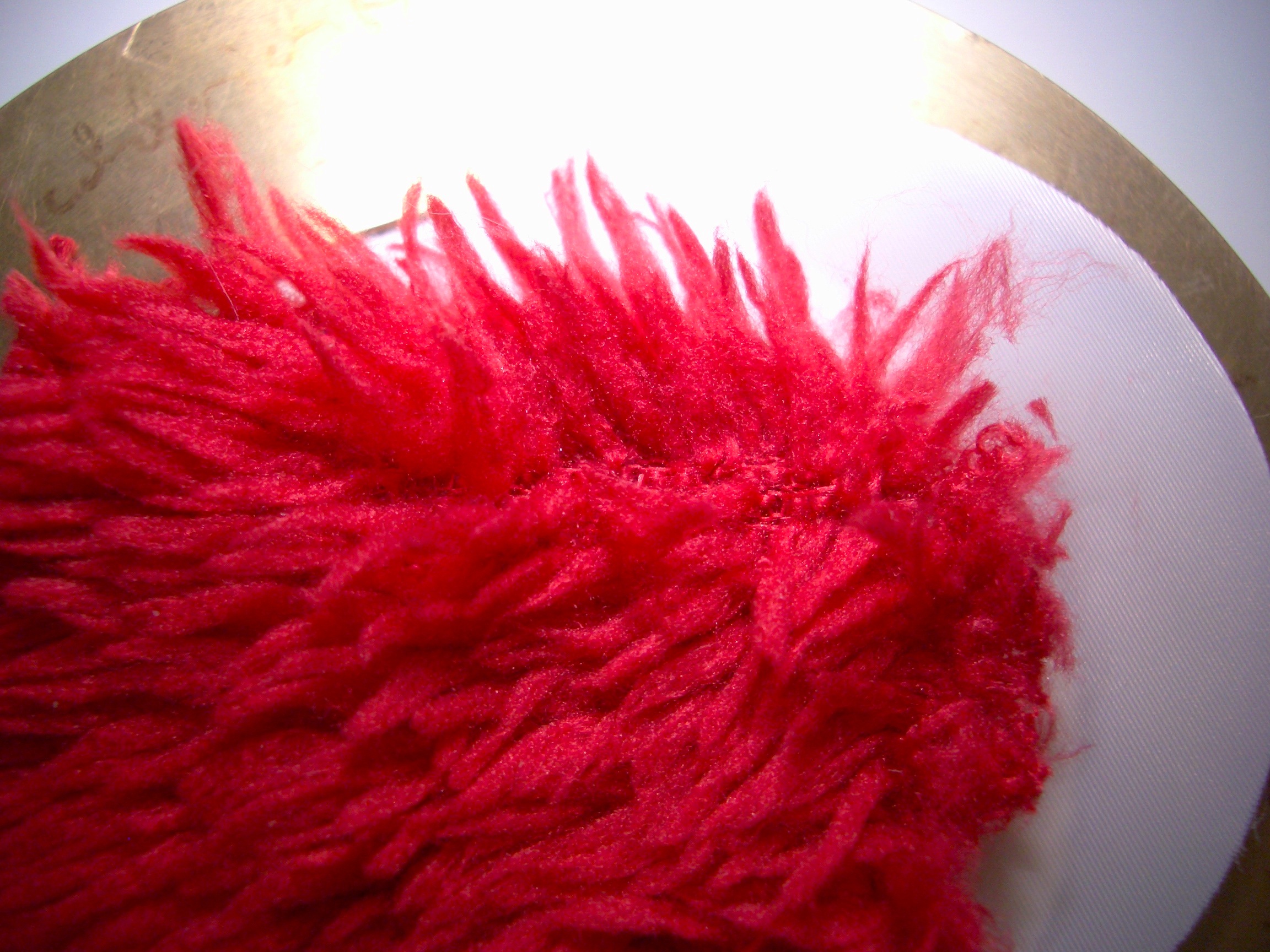

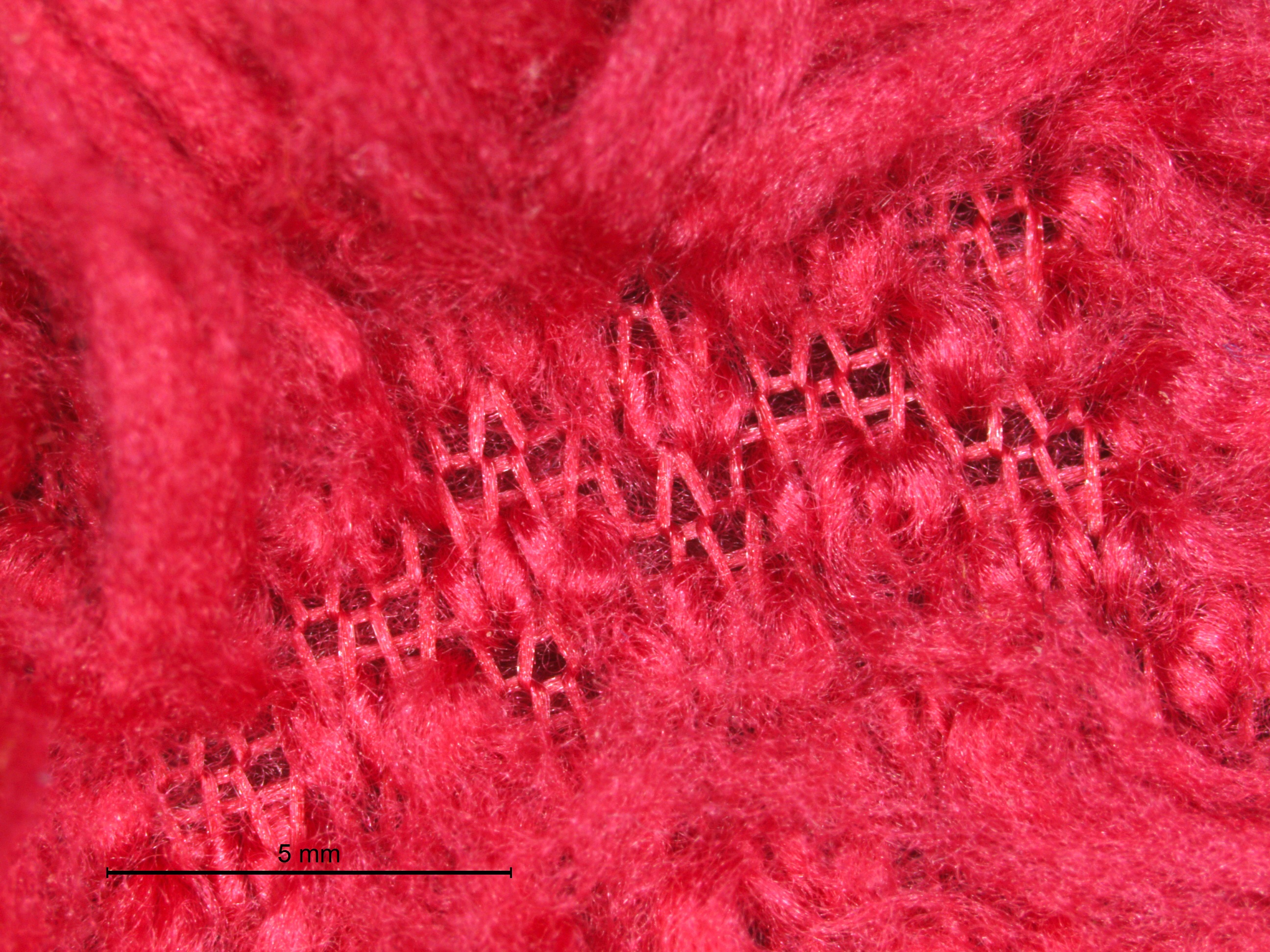

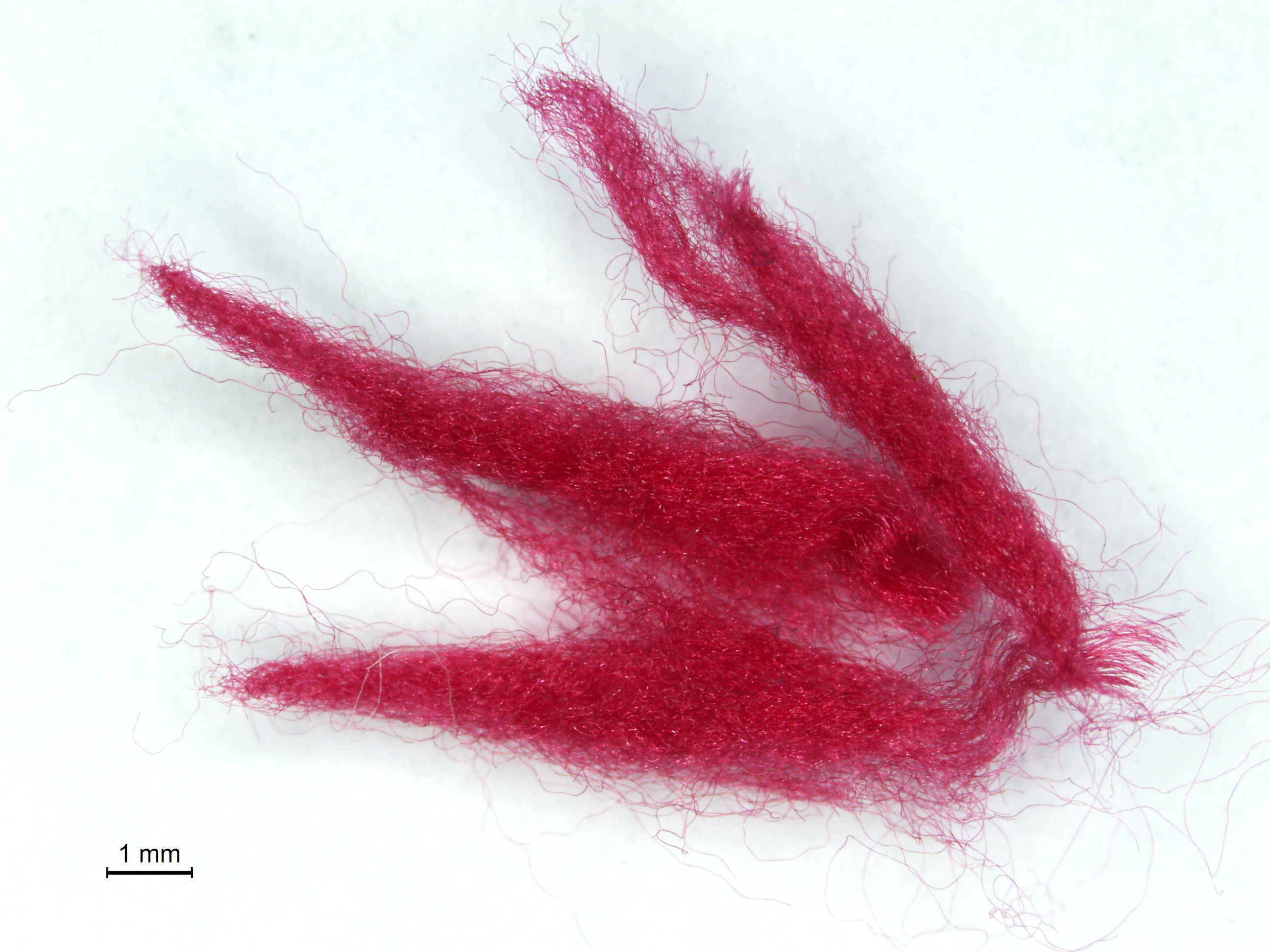

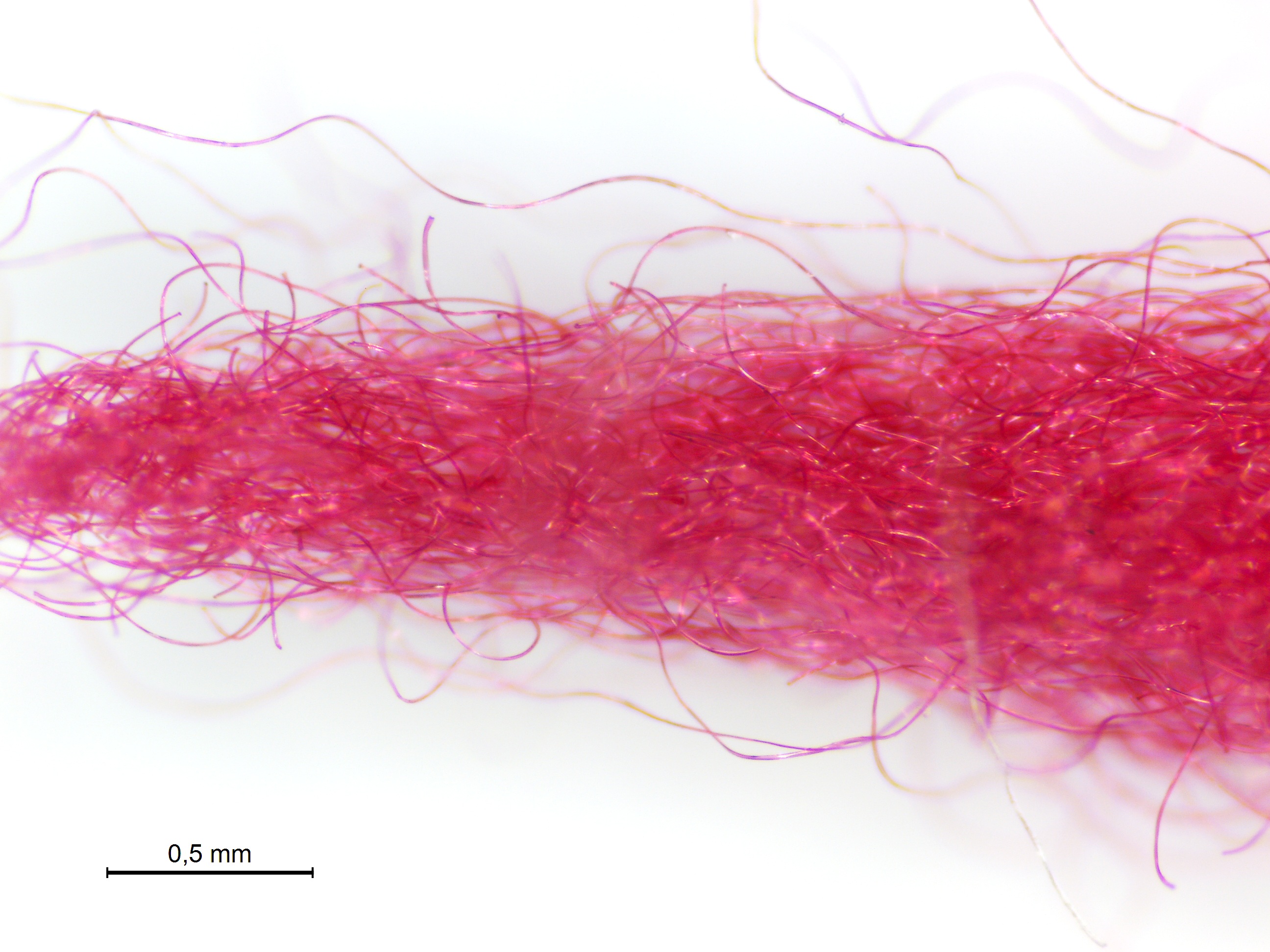


A

B

C

D

Figure S4. Stereo microscopy images of fabric structure: A blanket with visible piles, B ground-textile with piles, C microfiber piles, D detail of microfiber pile.


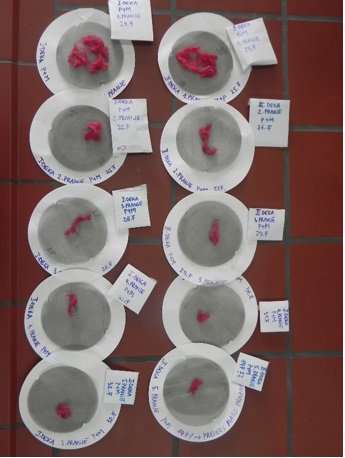

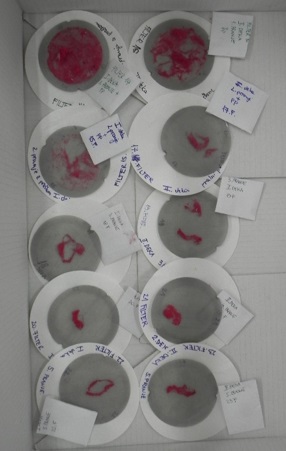

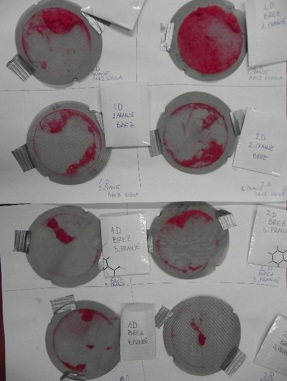


A

B

C

Figure S5. Photos of dried stainless steel filters with collected fibers after washing, with A) no additives (first four washing experiments), B) with detergent (first five washing experiments), C) with detergent and softener (first five washing experiments). Both parallel experiments (on two separate blankets) are shown in each case. It is visible that the quantity released in successive experiments decreases. The “fluffing” effect of softener is visible (series C vs. A and B).

Table S1. Fiber emissions from washing and drying in three series of experiments: i) no additives, ii) with washing detergent and iii) with washing detergent + softener (each series is comprised of 10 successive washing experiments done on two separate blankets (6 blankets in total). In total: 60 experiments/results. After each washing the blanket was tumble dried.

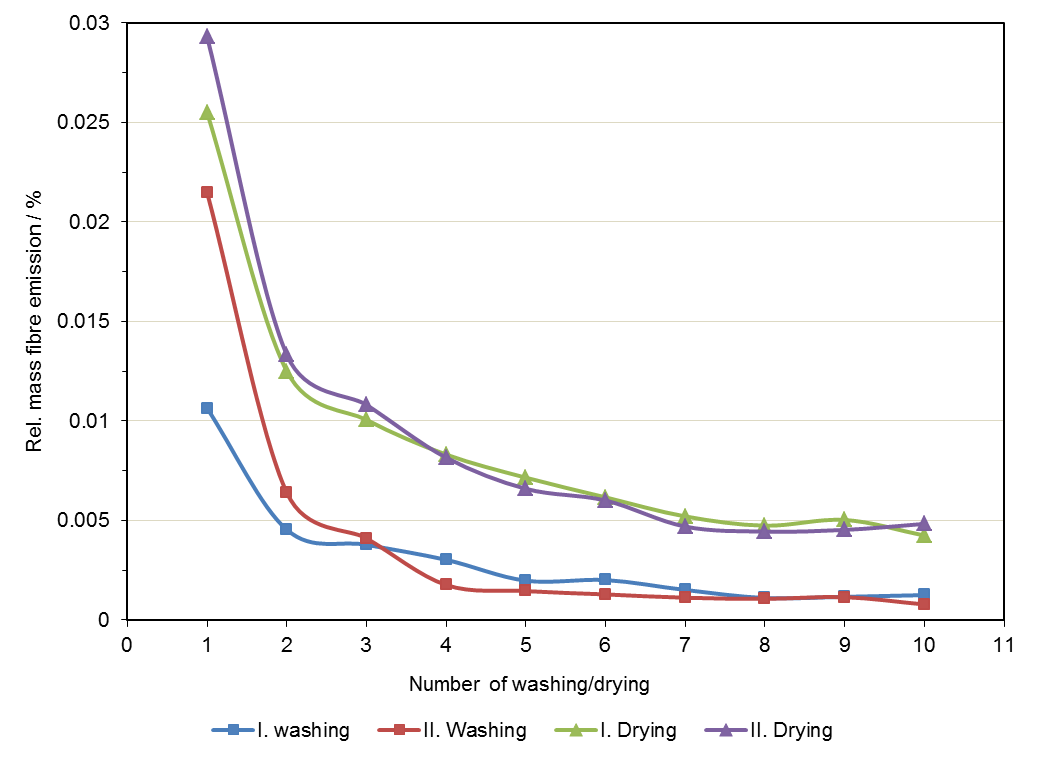

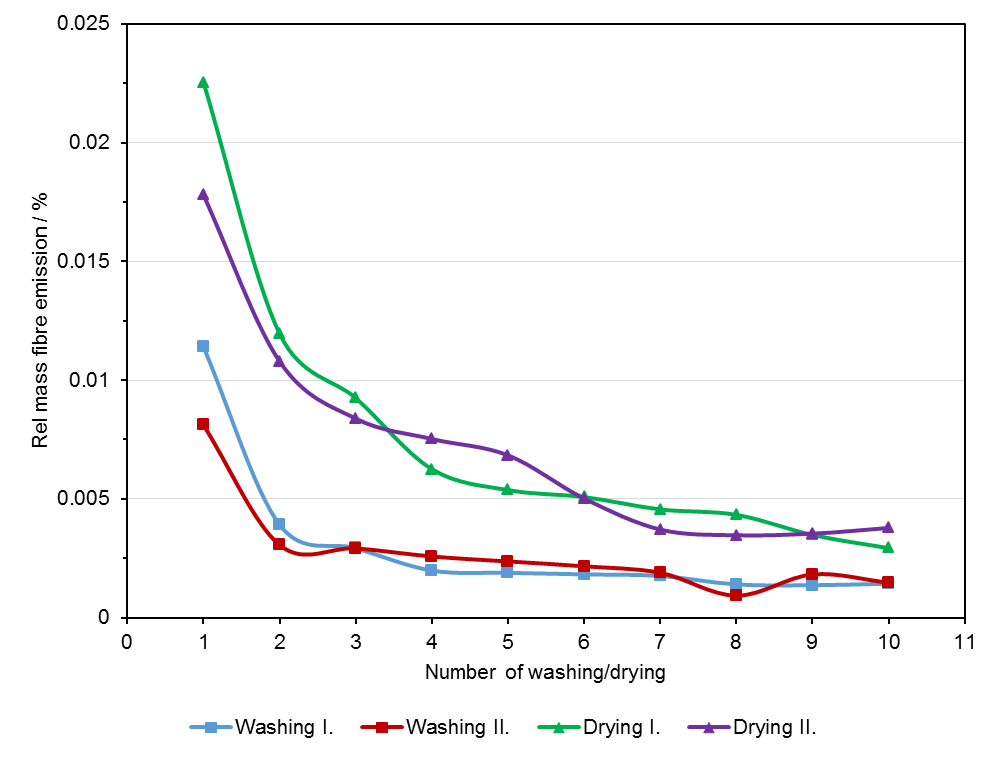

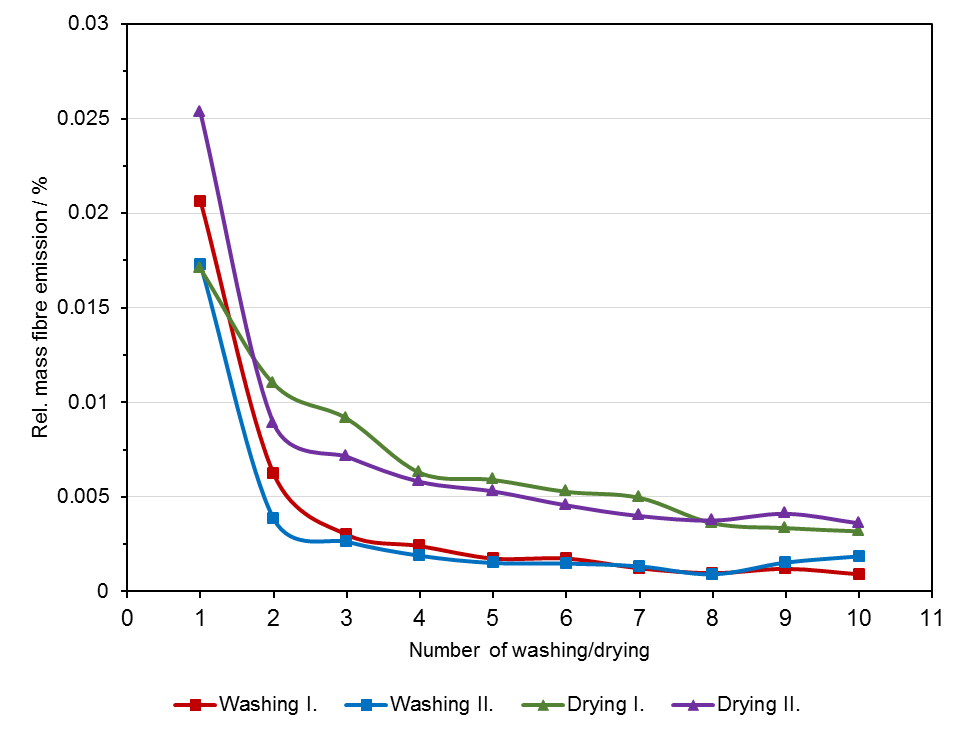


A

B

C

Figure S6. Plot of fiber emissions vs. successive washing/drying with A) no additives, B) with detergent, C) with detergent and softener (data from Table S1).


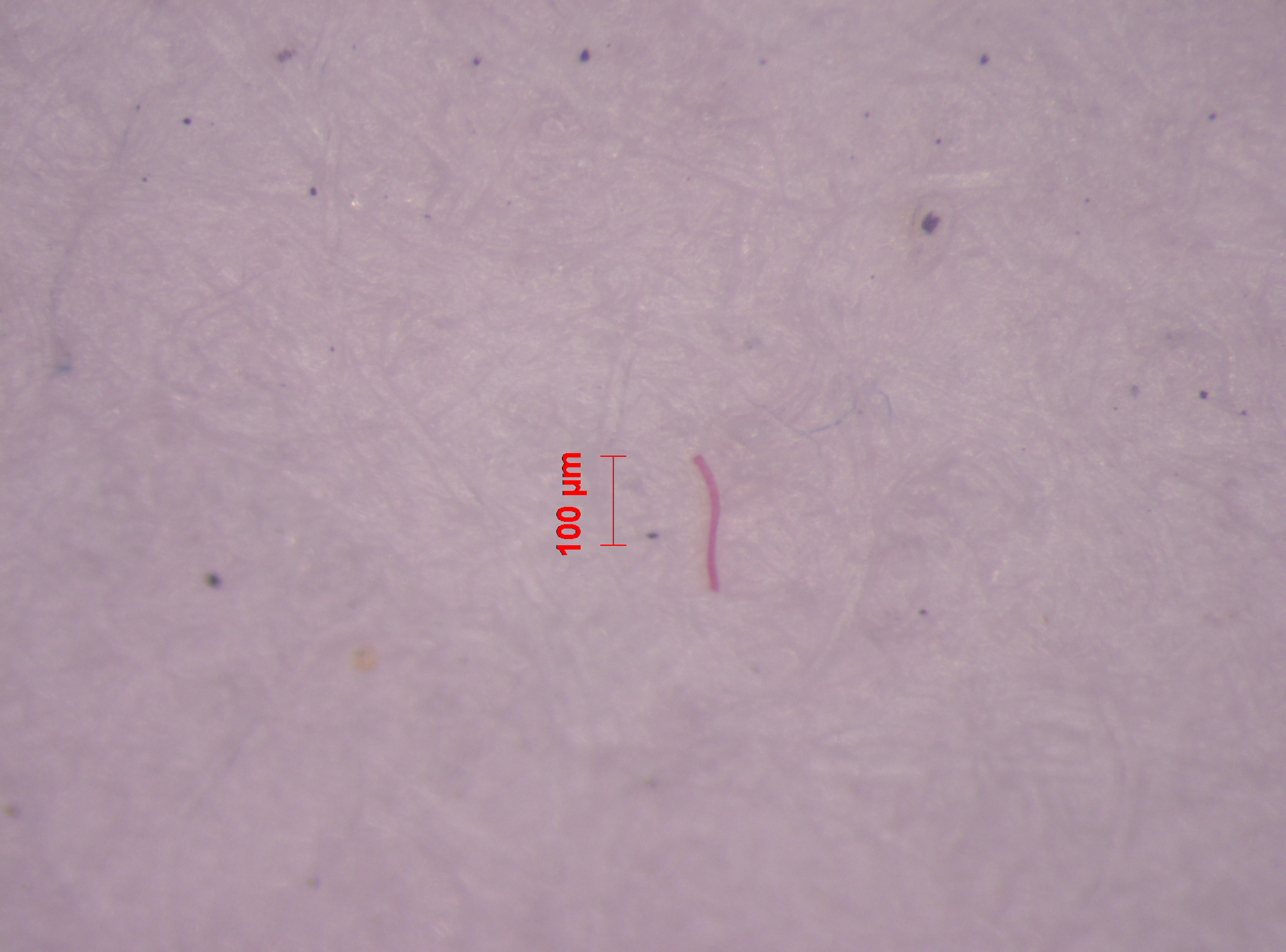


Figure S7. Stereo micrography of a representative short fiber that passed through the stainless steel filter. The size range of individual fibers was in the approx. size range 20 – 200 μm. The background is the paper filter.


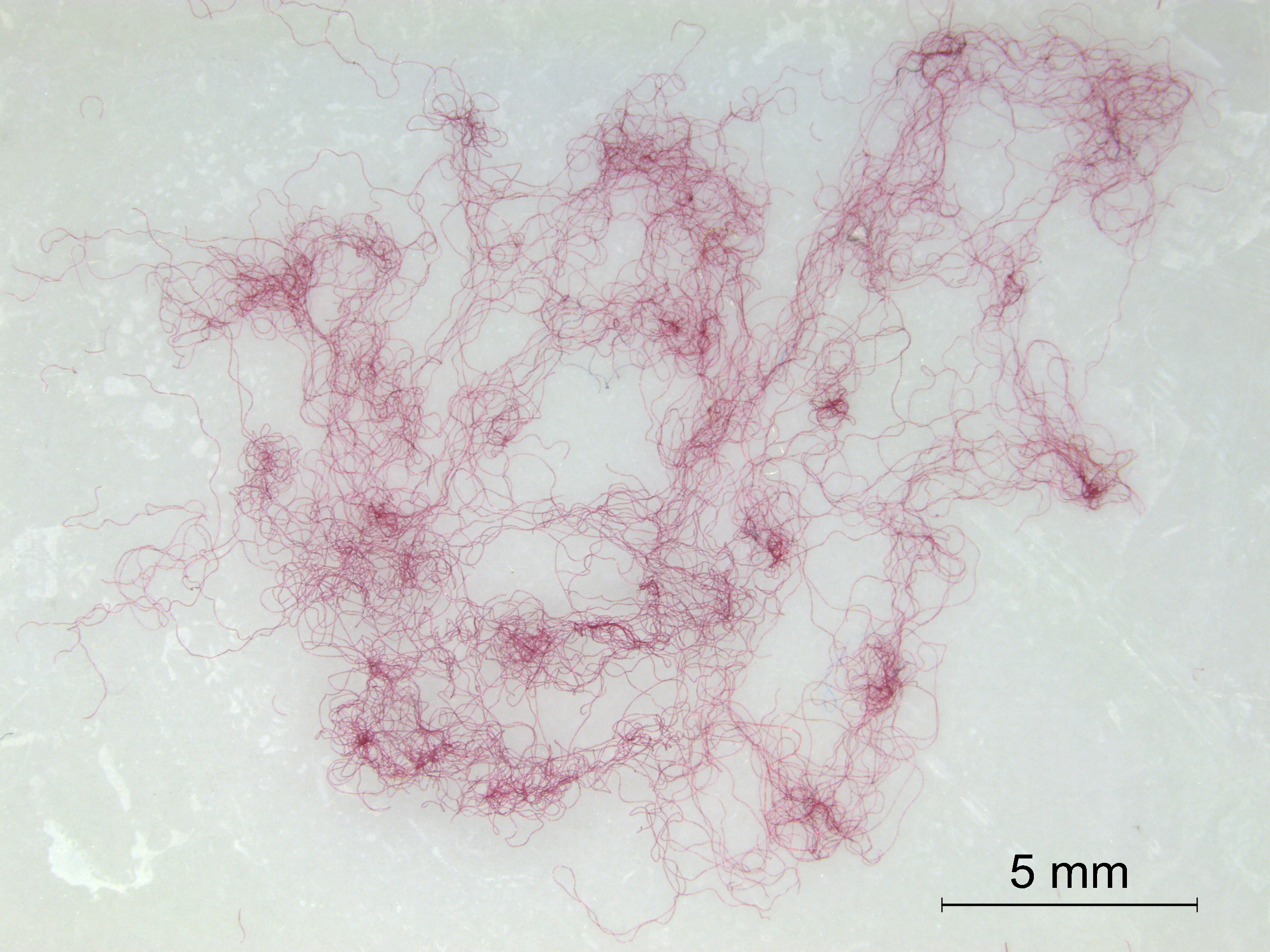

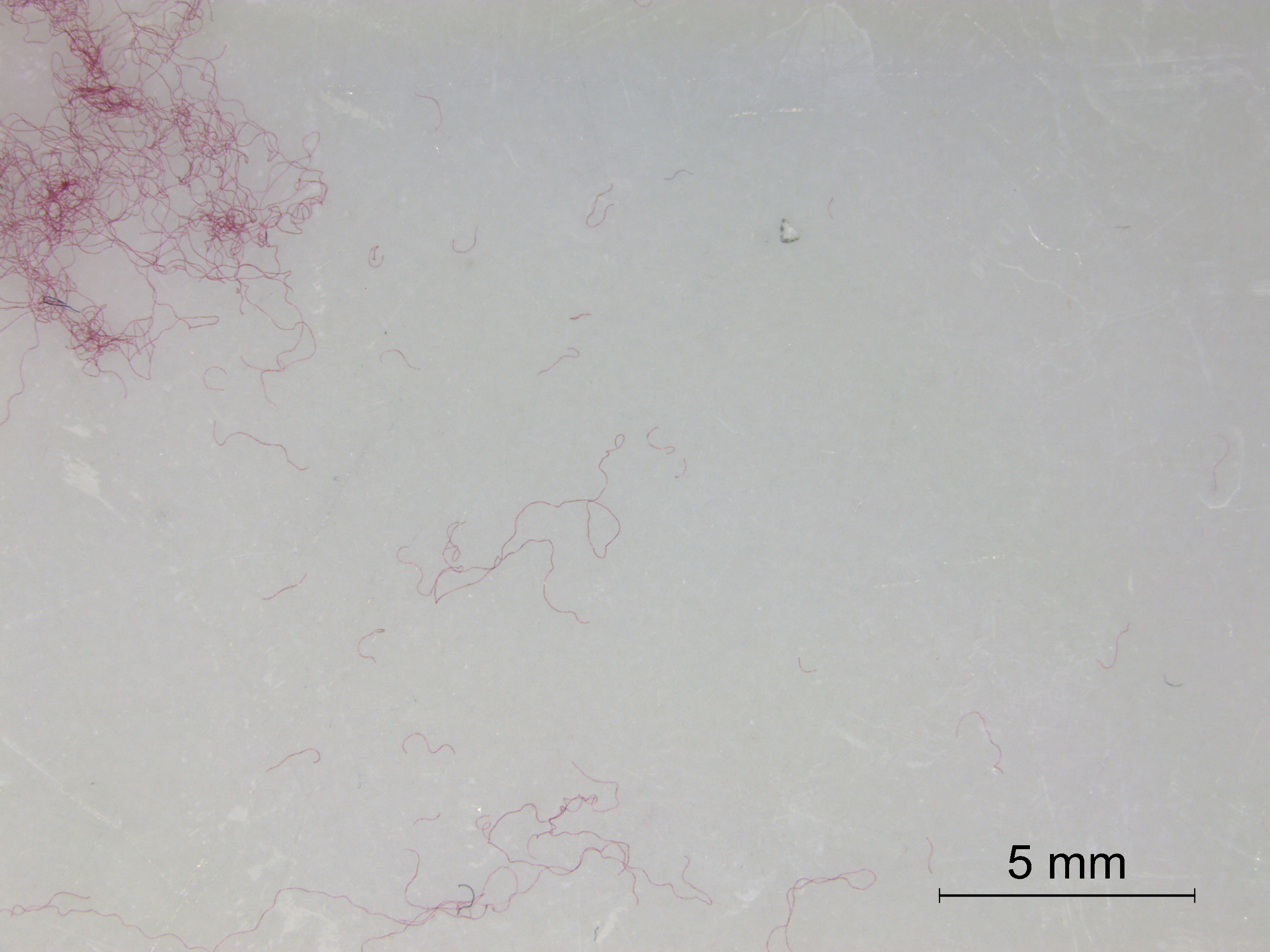

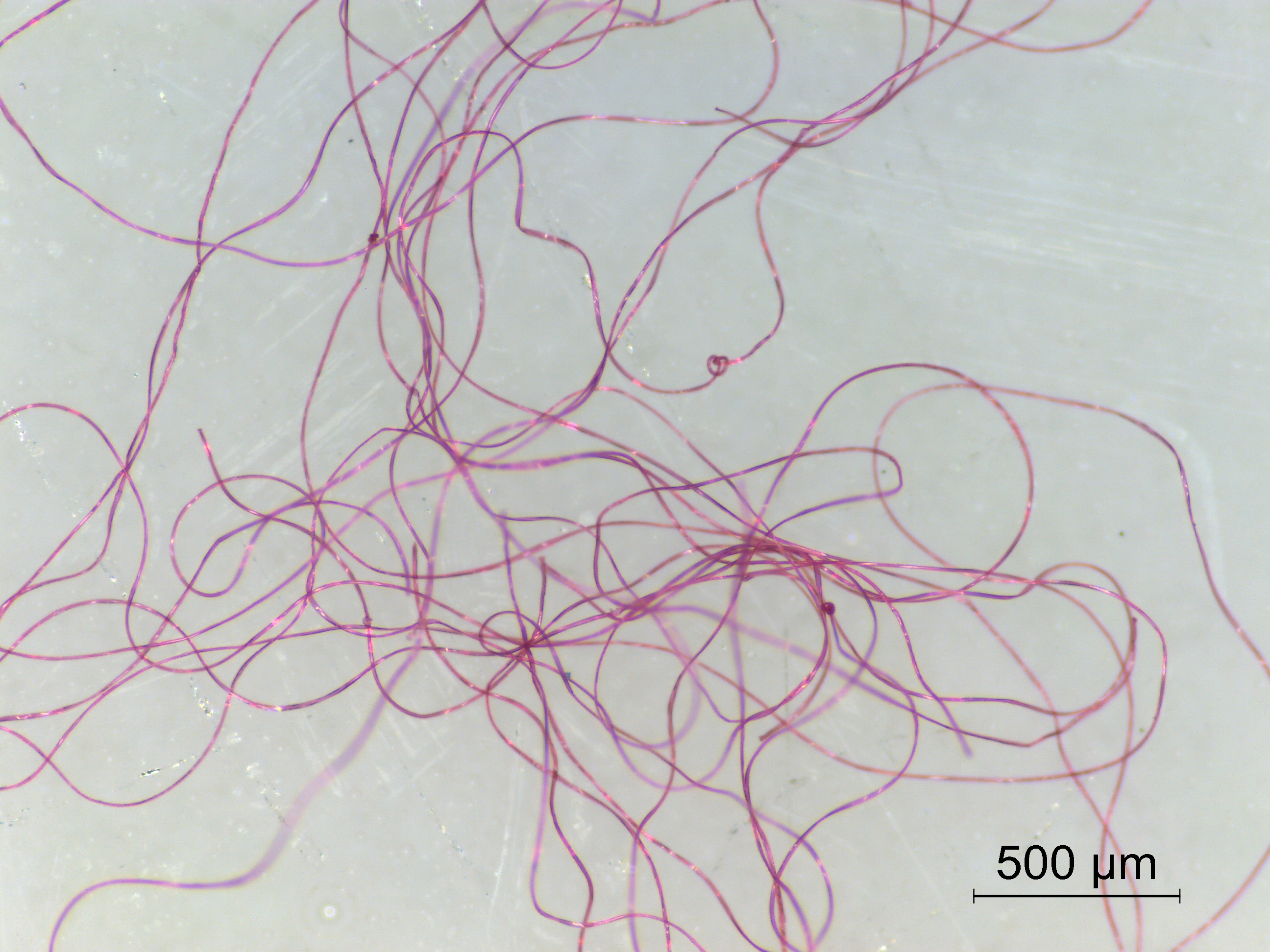

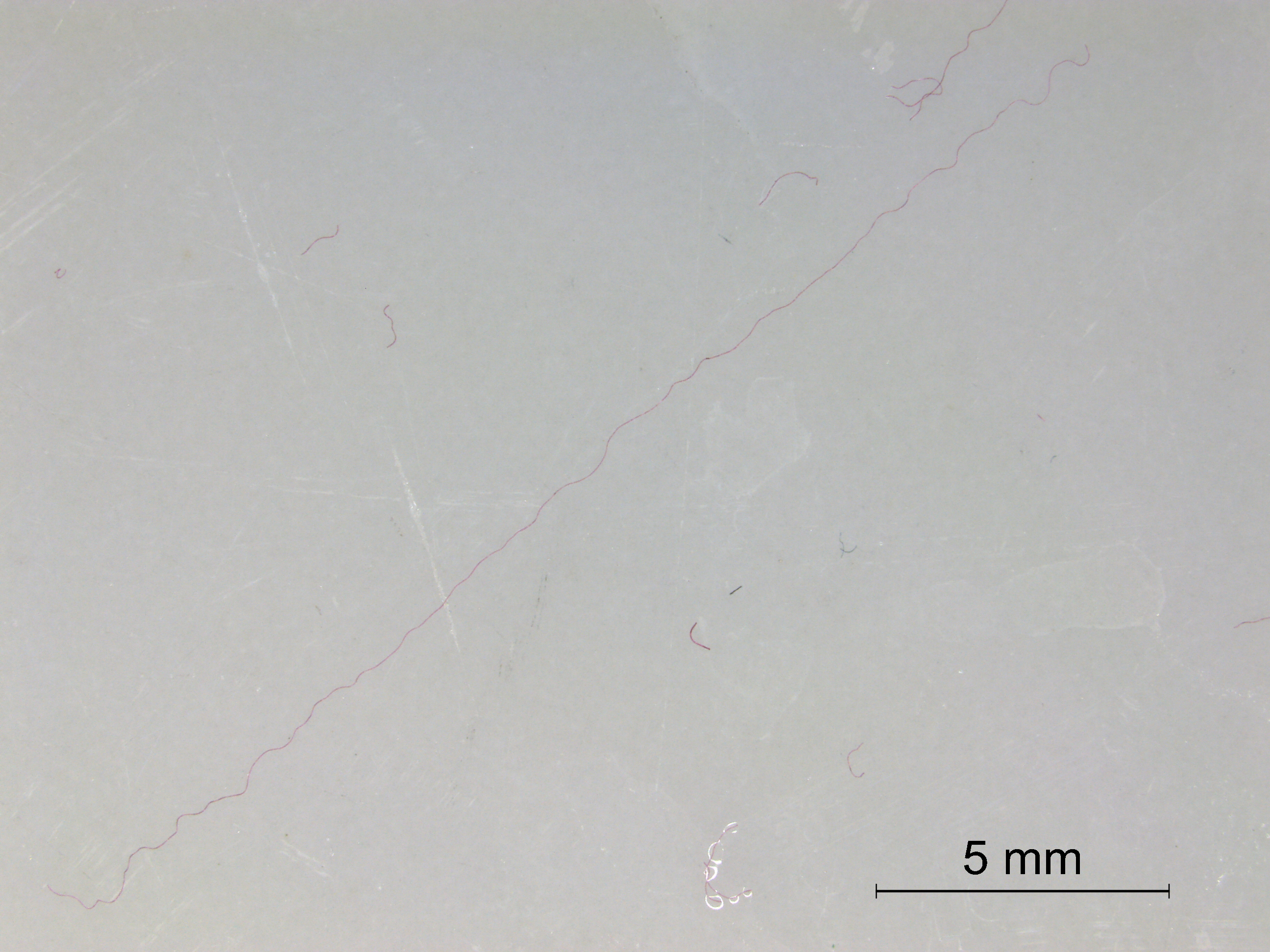


A

B

C

D

Figure S8. Stereo microscopy images of representative fibers collected on the 200 μm filter after the 5^th^ washing without additives. A) Partially spread out fibers remain strongly entangled. Intense entanglement of long fibers prevented performing a count of fibers. B) The small portion of short fibers visible after removing “fluff”. C) A very long fiber approx. 22 mm in length (stretched out for the purpose of measurement). This fiber represents the higher size limit of released fibers. D) Higher magnification showing detail of fiber entanglement.


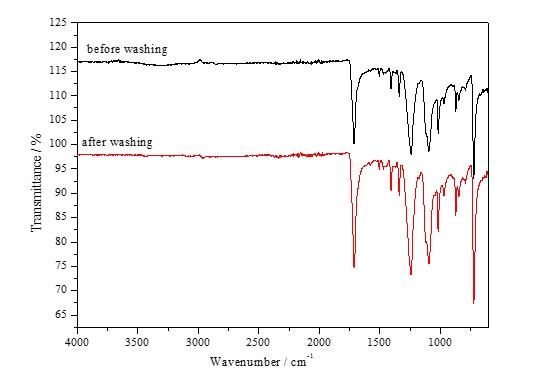


Figure S9. FTIR spectra of blanket fibers before and after washing and drying. Both IR spectra show absorptions characteristic for polyethylene terephthalate. The strong band at 1740 cm^-1^ is attributed to C=O stretching vibration mode of the ester bond.

1. Corresponding author; Contacts: telephone: +386-1-4760 296, fax: +386-1-4760 300, Email: andrej.krzan@ki.si [↑](#footnote-ref-1)
